# Alcohol consumption induces murine osteoporosis by downregulation of natural killer T‐like cell activity

**DOI:** 10.1002/iid3.485

**Published:** 2021-07-02

**Authors:** Munehiro Naruo, Yasuyuki Negishi, Takahisa Okuda, Midori Katsuyama, Ken Okazaki, Rimpei Morita

**Affiliations:** ^1^ Department of Microbiology and Immunology Nippon Medical School Tokyo Japan; ^2^ Department of Orthopaedic Surgery Tokyo Women's Medical University Tokyo Japan; ^3^ Department of Orthopaedic Surgery Tomei Atsugi Hospital Kanagawa Japan; ^4^ Department of Legal Medicine Nihon University School of Medicine Tokyo Japan; ^5^ Department of Legal Medicine Graduate School of Medical and Dental Sciences Kagoshima University Kagoshima Japan

**Keywords:** alcohol‐induced osteoporosis, dendritic cells, macrophages, NKT‐like cells, osteoclast

## Abstract

**Introduction:**

Chronic alcohol consumption (CAC) can induce several deleterious effects on the body, including the promotion of osteoporosis; however, the immunological mechanism underlying alcohol‐induced osteoporosis is still unclear.

**Methods:**

We administered alcohol to mice for 4 weeks as the experimental CAC model and analyzed the bone and immune cells that are located in the vicinity of a bone.

**Results:**

IL‐4 is known to be a suppressive factor for osteoclastogenesis, and we found that natural killer T (NKT)‐like cells, which showed NK1.1‐positive, CD3‐positive, and α‐galactosylceramide‐loaded CD1d tetramer‐negative, produced IL‐4 more effectively than CD4^+^ T and natural killer (NK) cells. The alcohol consumption facilitated a significant decrease of bone mineral density with the upregulation of nuclear factor of activated T cells 1 and receptor activator of NF‐κB ligand expression. Meanwhile, we confirmed that alcohol consumption suppressed the activity of antigen‐presenting cells (APCs) and NKT‐like cells, leading to decreased IL‐4 secretion. Moreover, these harmful effects of alcohol consumption were reduced by simultaneous treatment with a glycolipid antigen OCH.

**Conclusions:**

Our results indicate that the inactivation of innate immune cells, APCs, and NKT‐like cells are likely to be crucial for alcohol‐induced osteoporosis and provide a new therapeutic approach for preventing osteoporosis.

## Graphical abstract

We analyzed alcohol‐induced osteoporosis model mice, focusing on immune cells in bone. We mainly obtained three findings: (1) IL‐4‐highly producing NKT‐like cells were more abundant in bone, (2) oral alcohol consumption suppressed IL‐4 production from the NKT‐like cells, and (3) a glycolipid antigen OCH improved alcohol‐induced osteoporosis by restoring IL‐4 production.

## 1 INTRODUCTION

Excessive and chronic alcohol consumption (CAC) can cause harmful effects on the liver, heart, brain, and skeleton.^1^ Alcohol‐induced osteoporosis is a severe disorder induced by alcohol abuse and serves as a risk factor for osteopenia leading to bone fracture.^2^ Bone formation occurs via the balance between osteoblastogenesis and osteoclastogenesis. In CAC, this delicate balance is impaired, and alcohol‐induced osteoporosis develops. Osteoblasts originate from bone marrow mesenchymal stem cells (BMMSCs), and mature osteoblasts are entrapped in the bone matrix, in which they develop into osteocytes.^3^ Conversely, hematopoietic cells, namely macrophages/monocytes, in bone marrow (BM) differentiate into osteoclasts when several factors are present, such as cytokines, transcription factors, and receptor activator of NK‐κB ligand (RANKL).^4^, ^5^ The effects of alcohol on the differentiation and proliferation of osteoblasts, osteoclasts, and BMMSCs might involve pro‐inflammatory cytokines,^6^, ^7^, ^8^, ^9^, ^10^ Wnt/β‐catenin signaling,^11^, ^12^ mammalian target of rapamycin (mTOR) signaling,^13^ and oxidative stress.^14^ Moreover, recent studies in osteoimmunology suggested that several cytokines could modulate osteoclastogenesis. It has been generally accepted that the differentiation and proliferation of osteoclasts are suppressed by IFN‐γ and IL‐4 secretion by helper T (Th1) and Th2 cells.^15^, ^16^, ^17^, ^18^, ^19^ These findings indicate that immunological alterations in bone and BM by CAC may be crucial for the onset of alcohol‐induced osteoporosis. However, the precise mechanisms of the immune modulation of alcohol‐induced osteoporosis are not fully understood.

The mammalian immune system comprises the adaptive and innate immune systems. The former, which includes T and B cells, is characterized by high‐specificity antigen recognition with molecular rearrangement of receptors and immunologic memory. In contrast, the innate immune system, which includes macrophages, natural killer (NK) cells, dendritic cells (DCs), and NK T (NKT) cells, is unable to distinguish among specific proteins with minor differences in their structures; however, the innate immune system can respond rapidly to many antigen types.^20^, ^21^, ^22^ Antigen‐presenting cells (APCs), DC, and macrophages play important roles in protein antigen recognition and presentation to T cells via HLA class I and II molecules. In addition to processing and presenting protein antigens, APCs can also recognize damage‐associated molecular patterns and pathogen‐associated molecular patterns through pattern recognition receptors, leading to the secretion of pro‐inflammatory cytokines.^23^ NKT cells are a type of innate lymphocytes that are essential for host defense against cancer development, microbial infection, and reproduction.^21^, ^24^, ^25^, ^26^, ^27^, ^28^ NKT cells co‐express both T cell (CD3) and NK cell markers (NK1.1 in mice and CD56 in humans), and they can recognize lipid/glycolipid antigens presented by CD1d, a nonclassical MHC‐like glycoprotein, on APCs.^29^ NKT cells activated by APCs via the CD1d–TCR axis and secreted cytokines (e.g., IL‐12) can produce large amounts of IFN‐γ and IL‐4 rapidly.^21^, ^29^, ^30^, ^31^

In this study, we administered 10% ethanol/water solution to mice for 4 weeks to explore the effects of alcohol on bone loss in the early stage of CAC. We found that alcohol treatment can decrease the immunostimulatory activity of APCs and suppress NKT‐like cell activity, leading to the attenuation of IL‐4 secretion, which is a suppressive factor for osteoclastogenesis. Moreover, the sequential intraperitoneal administration of OCH, a glycolipid antigen, every 48 h prevented the decrease of bone loss induced by alcohol consumption via the stimulation of invariant NKT (iNKT) and NKT‐like cells. Understanding the immunological mechanisms of the regulation of osteoclastogenesis may provide new therapeutic approaches for alcohol‐induced osteoporosis.

## 2 MATERIALS AND METHODS

### 2.1 Mice

Female C57BL/6 (B6) mice were acquired from Charles River Laboratories. Female *Cd1d* ^−/−^ mice (NKT cell‐deficient mice) on a B6 background were acquired from the Jackson Laboratory. All mice were maintained in specific pathogen‐free conditions.

### 2.2 Alcohol consumption in mice

Nine‐week‐old female B6 and *Cd1d* ^−/−^ mice were assigned to treatment with alcohol or water. The alcohol‐treated group was administered 10% (w/v) ethanol/water solution ad libitum for 1 month as the sole drinking fluid. The concentration of the ethanol solution was increased gradient‐wise to 10% until 1 week before the initiation of the experiment. The mice in the water‐treated group were given only water for 4 weeks. The mice were fed commercial rodent food (MF pellets; Oriental Yeast Co., Ltd.) ad libitum in a specific pathogen‐free facility.

### 2.3 Administration of OCH, anti‐IL‐4 antibody, and anti‐IFN‐γ antibody to alcohol‐treated B6 mice

Alcohol‐treated B6 mice received intraperitoneal injections of OCH (2 μg/mouse, dissolved in dimethyl sulfoxide and diluted with phosphate‐buffered saline [PBS]; Adipogen Life Sciences) or vehicle every 48 h from 9 to 13 weeks of age. Mice were euthanized 24 h after the last dose of OCH or vehicle, and bone mineral density (BMD), messenger RNA (mRNA) expression, and intracellular cytokine production were evaluated. Anti‐IL‐4 neutralizing antibody (Biogems), anti‐IFN‐γ neutralizing antibody (Biogems), and control IgG (rat IgG1 kappa monoclonal; Abcam plc) was also administered to alcohol‐treated B6 mice at the same time as OCH every 48 h. These mice were also euthanized 24 h after the last dose of OCH and BMD.

### 2.4 Trabecular and cortical BMD measured using quantitative computed tomography (qCT)

At 13 weeks of age, mice were euthanized via cervical dislocation, and tibiae were separated for qCT. Isolated left tibiae were scanned using a peripheral qCT system (LaTheta LCT‐200; Hitachi Aloka Medical) as previously described.^32^ Briefly, the configuration was as follows: a voxel size of 24 × 96 μm at a tube voltage of 50 kVp, a tube current of 0.5 mA, an integration time of 26 ms for each projection, and an axial field view of 200 mm. The region of interest for trabecular BMD was defined as 1 mm distal from the growth plate and that for cortical BMD was defined as the center of diaphysis using Latheta software version 3.44 (Hitachi Healthcare).

### 2.5 Bone morphology analysis by micro‐computed tomography (μCT)

After the cervical dislocation, tibiae of 10% ethanol‐treated or water‐treated mice were separated for μCT. Trabecular bone morphology of proximal tibia was measured using an ex vivo μCT device (Scan Xmate‐L090H; Comscasntecno, Co., Ltd.). The X‐ray settings were standardized to 74 kV, 100 μA with a magnification power of 23.2134, the resolution of 10.942; μm/pixel, and the slice thickness of 10.942 μm. The bone morphological parameters of bone volume/total tissue volume (BV/TV) (%) were calculated on 2D images by using an image analysis software (TRI/3‐D‐BON; Ratoc System Engineering, Co., Ltd.), followed by coronal plane reconstruction.

### 2.6 Tartrate‐resistant acid phosphatase (TRAP) staining

Tibiae of 10% ethanol‐treated or water‐treated mice were also separated for TRAP staining. The samples were fixed in 4% paraformaldehyde at 4°C for 48 h, followed by immersion fixation in 30% sucrose at 4°C for 48 h. Next, the samples were placed in the Tissue‐Tek O.C.T. compound (SAKURA) at −80°C until staining. The cryostat section (8 μm) was obtained by Kawamoto's film method (SECTION‐LAB, Co., Ltd.)^33^ and stained using the TRAP Kit (FUJIFILM Wako Pure Chemical Corporation) according to the manufacturer's protocol.

### 2.7 Quantification of mRNA using quantitative reverse‐transcription polymerase chain reaction (RT‐qPCR)

Femurs obtained from B6 and *Cd1d* ^−/−^ mice were frozen with liquid nitrogen and crushed into fragments to create a lysate. Total RNA was extracted from the lysate (30 mg) using a Qiagen RNeasy Fibrous Tissue Mini Kit (Qiagen) with DNase digestion. Up to 2 µg of mRNA were reverse‐transcribed using a High‐Capacity cDNA Reverse Transcription Kit (Applied Biosystems) according to the manufacturer's instruction. RT‐qPCR was performed using Fast SYBR Green PCR Master Mix (Life Technologies) on an ABI 7500 Fast Real‐Time PCR System (Applied Biosystems) as previously described.^32^ The gene‐specific primers designed by Primer‐3‐Plus (http://primer3plus.com) are listed in Table S1. Target mRNA amplification was confirmed by the normalized threshold cycle values according to the β‐actin mRNA level. All data were analyzed using ABI 7500 Software version 2.3 (Applied Biosystems).

### 2.8 Cell preparation and flow cytometry

To examine immune cells in bone, BM was roughly removed from femurs and tibiae; then, we assessed the immune cells in bone that loosely adhered in cancellous and cortical bone. The femurs and tibiae were cut into small pieces with ophthalmic scissors. Subsequently, the pieces were suspended in RPMI‐1640 medium (Sigma‐Aldrich) and incubated with 1 mg/ml collagenase D (Roche) at 37°C for 30 min. The pieces of bone were smashed roughly using a sterile mortar and incubated with 1 mg/ml collagenase D at 37°C for an additional 60 min. After passing through a nylon mesh, mononuclear cells were purified using Lympholyte (Cedarlane) as per the manufacturer's instructions. After washing twice with PBS (pH 7.4), the cells were incubated with an Fc blocker (clone 24G2; American Type Culture Collection) for 15 min to block the nonspecific binding of mononuclear antibodies (mAbs) with Fc receptors. After washing twice with buffer, the cells were stained with anti‐mouse mAbs at 4°C for 30 min. The mAbs used in the present study are described in Table S2. After washing twice with buffer, the stained cells were analyzed using an LSRFORTESSA X‐20 flow cytometer (Becton Dickinson Immunochemical System) and FlowJo software (Tree Star, Inc.). The gating strategy of immune cells is presented in Figure S2.

For the analysis of immune cells in BM obtained from femurs and tibiae, BM was suspended in RPMI and passed through a nylon mesh. Then, mononuclear cells were purified using Lympholyte and incubated with an Fc blocker. Next, the cells were stained with mAbs and analyzed using flow cytometry. The gating strategy of immune cells is presented in Figure S3.

To analyze immune cells in the spleen, the spleen was carefully separated from mice and cut into small pieces with ophthalmic scissors. The tissues were incubated with 1 mg/ml collagenase D at 37°C for 45 min. Then, the tissues were mashed finely and passed through a nylon mesh. Mononuclear cells were isolated as described for cells in BM and bone, and cells were analyzed via flow cytometry. The gating strategy of immune cells is presented in Figure S4.

### 2.9 Detection of intracellular cytokines and transcription factors

The mononuclear cells in bone obtained from the alcohol‐ and water‐treated mice were incubated with anti‐mouse mAbs (anti‐CD3 mAb, anti‐CD45 mAb, anti‐CD19 mAb, anti‐NK1.1 mAb, and α‐GalCer‐loaded CD1d tetramer) for extracellular staining, as described previously. The mice were injected with 250 μg of brefeldin A intraperitoneally 4 h before euthanasia. A Cytofix/Cytoperm Kit (BD Pharmingen) was applied for intracellular staining. The fluorescent antibodies employed for the intracellular cytokine detection were PE‐conjugated anti‐IL‐4 mAb, PE‐conjugated anti‐IFN‐γ mAb, and PE‐conjugated anti‐IL‐12 mAb. We evaluated IL‐4 and IFN‐γ production by NKT‐like, NK, and CD4^+^ T cells. IL‐12 production by DCs and macrophages was also determined. PE‐conjugated anti‐T‐bet mAb and PE‐conjugated anti‐GATA3 mAb were used to detect intracellular transcriptional factors. A Zombie Violet FixableViability Kit (BioLegend) was used to eliminate dead cells according to the manufacturer's protocol. The details of the mAbs employed in this study are presented in Table S2.

### 2.10 Statistical analysis

The nonparametric Mann–Whitney *U* test was performed for comparisons between groups. For comparisons among more than two groups, a one‐way analysis of variance followed by Bonferroni post‐tests was performed. The results are presented as box plots with medians and interquartile ranges or as the mean ± *SD*. Results were considered significantly different at *p* < .05. All statistical analyses were performed using Prism (GraphPad Software).

## 3 RESULTS

### 3.1 Alcohol consumption reduces BMD of trabecular and cortical bone with the upregulation of Nfatc1 and Rankl

**Figure 1:**
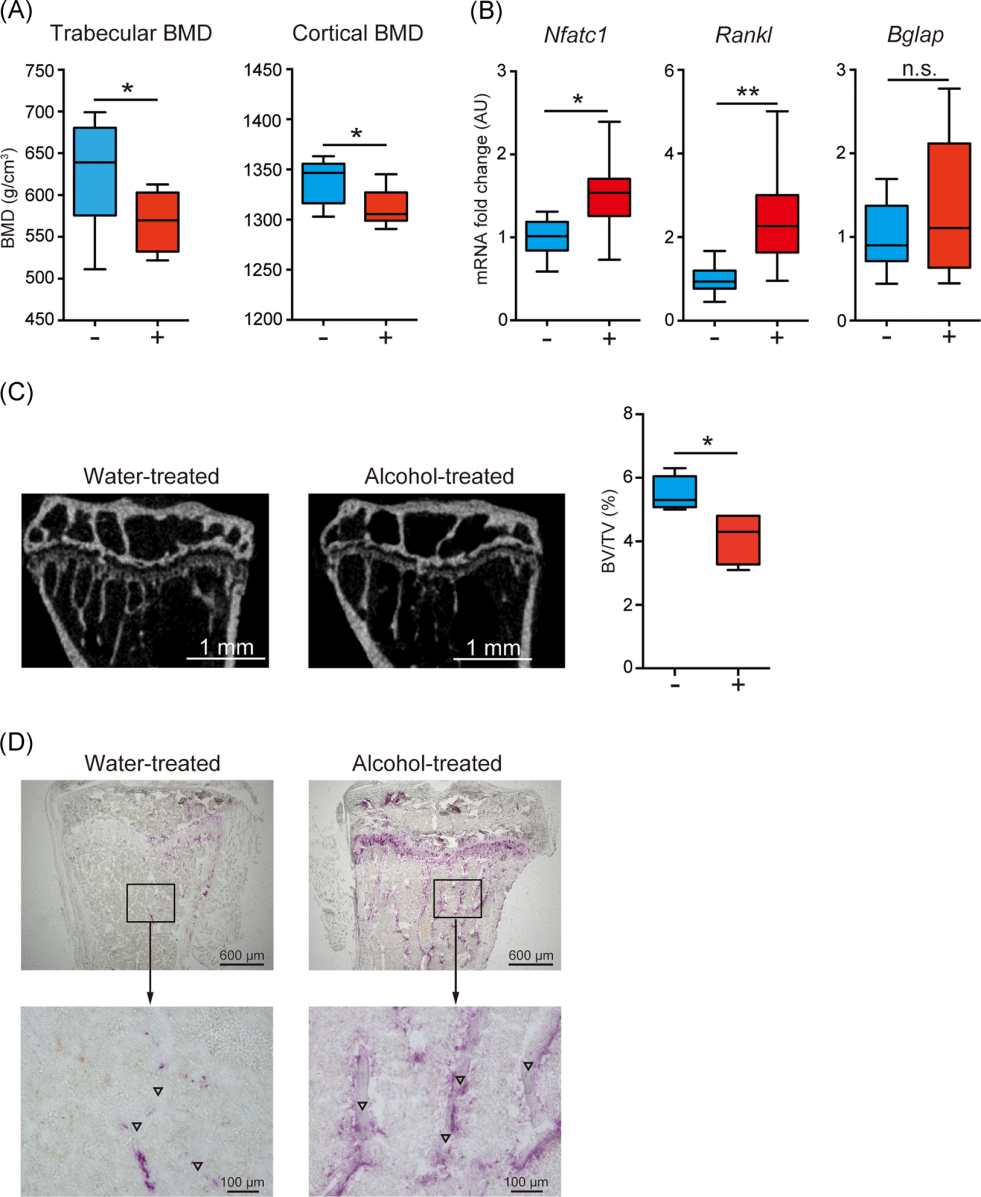
Alcohol consumption induces bone loss with the upregulation of genes related to osteoclast differentiation. (A) Trabecular and cortical bone mineral density (BMD) in alcohol‐treated (red) and water‐treated (blue) B6 mice, as measured using quantitative computed tomography. (B) Messenger RNA expression of *Nfatc1*, *Rankl*, and *Bglap* in bone from alcohol‐treated (red) and water‐treated (blue) B6 mice. (C) The representative feature of tibia treated with water and 10% ethanol (left), and the bone volume/total tissue volume (BV/TV) ratio (right) evaluated using micro‐computed tomography. (D) Tartrate‐resistant acid phosphatase staining of mice tibia treated with water (left panel) and 10% ethanol (right panel). Osteoclasts are represented in purple, (▽) indicates the bone matrix. Box plots: horizontal lines of boxes represent the medians, the boxes represent the interquartile ranges, and the whiskers extend to extreme values (8 mice/group). The data were pooled from six independent experiments. *p < .05, **p < .01; Mann–Whitney *U* test. n.s., not significant

BMD of trabecular and cortical bone was evaluated in alcohol‐treated (10% [w/v] ethanol/water solution given ad libitum for 1 month) and water‐treated B6 mice via qCT. Both trabecular and cortical BMD were significantly decreased by alcohol consumption (Figure 1A). These results indicate that alcohol consumption for 4 weeks induces osteoporosis morphologically in our system. To clarify bone metabolic turnover at the gene level, RT‐qPCR was performed in mice from both groups. Nuclear factor of activated T cells 1 (*Nfatc1*) and *Rankl*, which are involved in osteoclast differentiation, were overexpressed in alcohol‐treated B6 mice compared with their levels in water‐treated B6 mice, whereas the expression of the bone gamma‐carboxyglutamate protein (*Bglap*) was not different, which reflects osteoblast differentiation activity (Figure 1B). These results indicated that alcohol consumption could induce osteoclastogenesis at the gene level. We found that the structure of trabecular treated with alcohol was sparse when compared to that treated with water using μCT analysis (Figure 1C, left panel). The BV/TV ratio decreased in alcohol‐treated B6 mice (Figure 1C, right panel). Moreover, TRAP staining, which stains the osteoclasts, was performed for mice tibia treated with water and 10% ethanol. The osteoclasts were abundant in alcohol‐treated mice when compared with water‐treated mice (Figure 1D, upper lane). In particular, the osteoclasts were located around the bone matrix in alcohol‐treated mice (Figure 1D, lower lane). Collectively, alcohol consumption could induce bone loss with the accumulation of osteoclasts and upregulation of *Nfatc1* and *Rankl*.

### 3.2 NKT‐like cells are abundant in bone and are the major source of IL‐4

**Figure 2:**
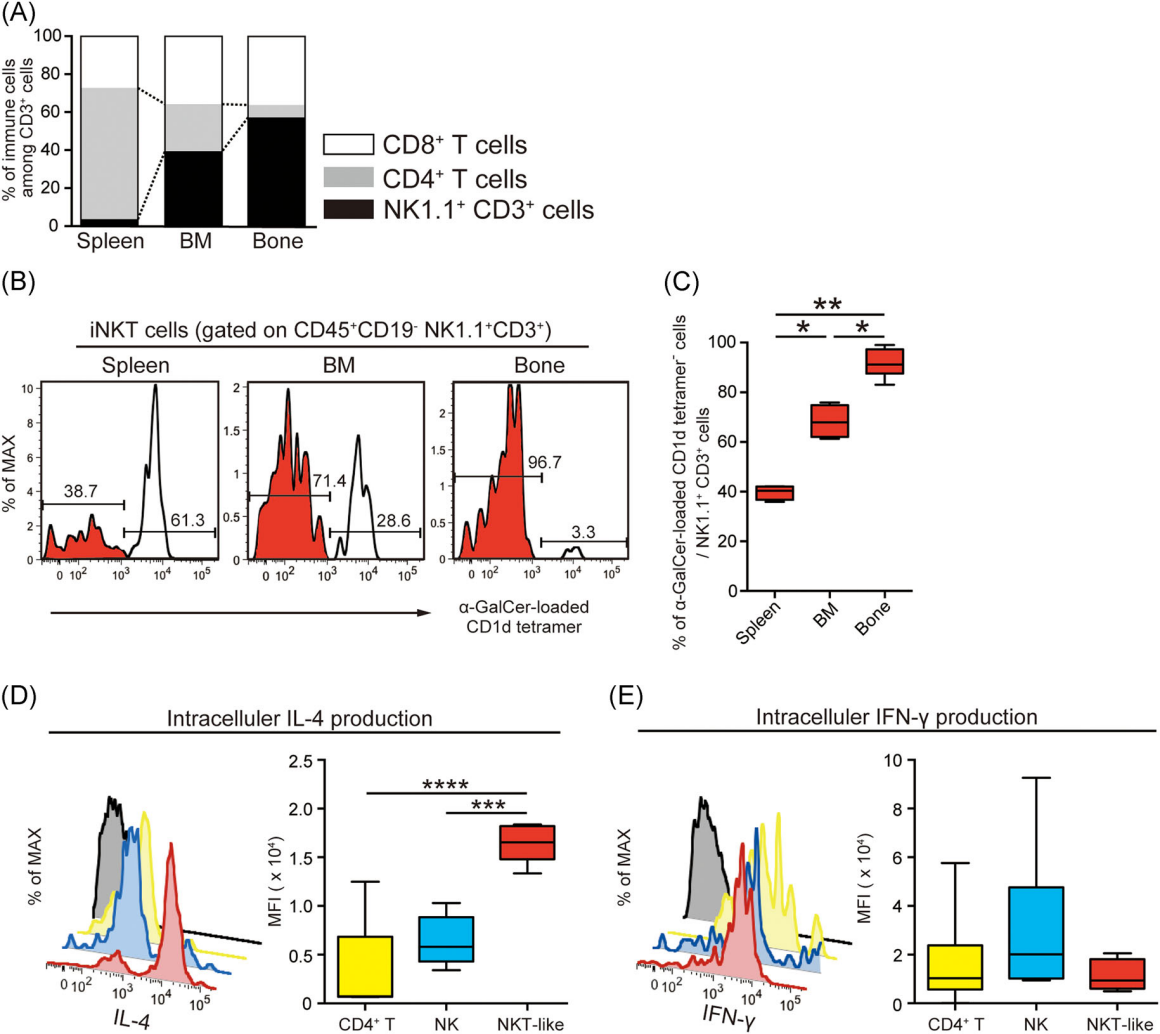
Natural killer T (NKT)‐like cells are abundant in bone and are a major producer of IL‐4. (A) The percentages of CD8^+^ T cells, CD4^+^ T cells, and NK1.1^+^CD3^+^ cells among viable CD3^+^ cells. The values indicate the average of seven mice per group. (B, C) The percentage of α‐GalCer‐loaded CD1d tetramer‐negative cells among viable NK1.1^+^CD3^+^ cells (red) in the spleen, bone marrow (BM), and bone (7 mice/group). The data were pooled from six independent experiments (A–C). (D, E) Comparison of intracellular IL‐4 and IFN‐γ levels among CD4^+^ T cells (yellow), NK cells (blue), and NKT‐like cells (red) in bone for water‐treated B6 mice. Cytokine production was evaluated by flow cytometry after intracellular staining (5–6 mice/group). The data were pooled from five independent experiments. Box plots: horizontal lines of boxes represent the medians, the boxes represent the interquartile ranges, and the whiskers extend to extreme values. Gray, isotype control. *p < .05, **p < .01, ***p < .005, ****p < .001; one‐way analysis of variance followed by Bonferroni post‐tests

The populations of macrophages, DCs, CD8^+^ T cells, CD4^+^ T cells, NK1.1^+^CD3^+^ cells, and NK cells in the spleen, BM, and bone were determined using flow cytometry. The percentage of macrophages was higher in BM and bone than in the spleen (Figure S1). Conversely, the percentages of CD8^+^ and CD4^+^ T cells were lower in BM and bone than in the spleen (Figure S1). The gating strategies of representative flow cytometric plots are presented in Figures S2–S4. We assessed the percentages of CD8^+^ cells, CD4^+^ T cells, and NK1.1^+^CD3^+^ cells among CD3^+^ cells. The percentage of NK1.1^+^CD3^+^ cells was higher than those of CD8^+^ T cells and CD4^+^ T cells in BM and bone (Figure 2A). Hence, the percentage of NK1.1^+^CD3^+^ cells among CD3^+^ T cells was different between the bone and spleen.

In general, type I NKT cells are known as invariant NKT (iNKT) cells that are detected by α‐galactosylceramide (α‐GalCer)‐loaded CD1d tetramer positivity.^30^ The percentage of iNKT cells was substantially lower in bone than in BM and the spleen (Figure 2B,C). Therefore, we regarded NK1.1‐positive, CD3‐positive, and α‐GalCer‐loaded CD1d tetramer‐negative cells as NKT‐like cells in this study and mainly focused on these cells, which are located in the vicinity of bone and which can be expected to more strongly contribute to bone remodeling.

IL‐4 and IFN‐γ inhibit the development of osteoclasts.^15^, ^16^, ^18^ In general, these cytokines are primarily produced by CD4^+^ T cells, NK cells, and NKT cells. Therefore, we examined IL‐4 and IFN‐γ production by CD4^+^ T cells, NK cells, and NKT‐like cells in bone. NKT‐like cells were the most powerful producers of IL‐4 (Figure 2D). IFN‐γ production did not significantly differ among these cells (Figure 2E). These results indicate that NKT‐like cells could have significant effects on bone resorption.

Next, we measured the percentage of immune cells in bone obtained from alcohol‐ or water‐treated B6 mice (Figure S5). The populations of macrophages, DCs, CD8^+^ T cells, CD4^+^ T cells, NK cells, and NKT‐like cells were not significantly different between alcohol‐ and water‐treated B6 mice.

### 3.3 Alcohol consumption suppresses IL‐4 production of NKT‐like cells with the reduction of immunostimulatory activity of macrophages and DCs

**Figure 3:**
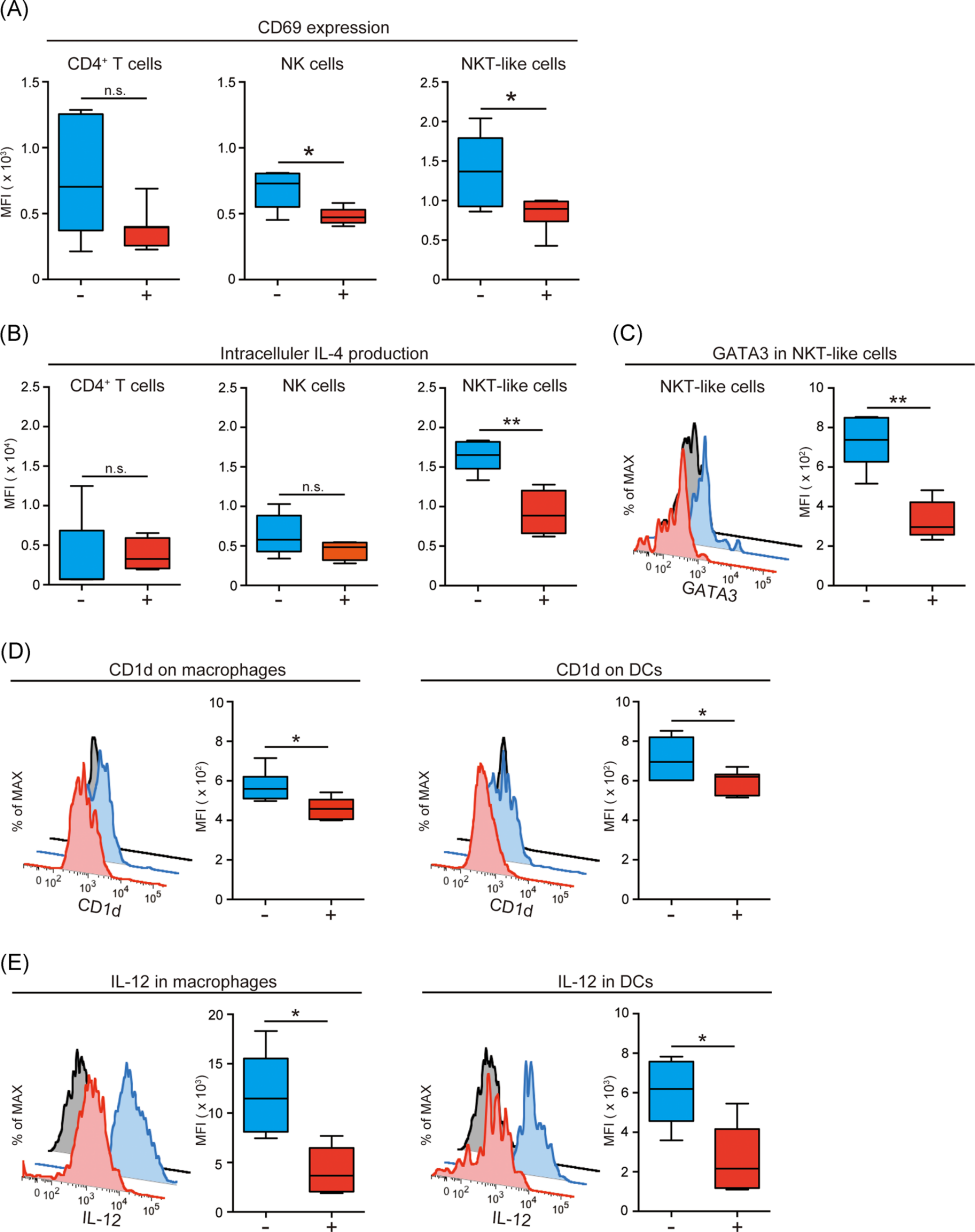
Alcohol consumption suppresses IL‐4 production of natural killer T (NKT)‐like cells with the reduction of immunostimulatory activity of antigen‐presenting cells. (A) CD69 expression on CD4^+^ T cells, NK cells, and NKT‐like cells in bone analyzed by flow cytometry (6–8 mice/group). The data were pooled from six independent experiments. (B) Intracellular IL‐4 expression on CD4^+^ T cells, NK cells, and NKT‐like cells in bone analyzed by flow cytometry (5–6 mice/group). The data were pooled from five independent experiments. (C) The expression of GATA3 in NKT‐like cells analyzed by flow cytometry (5 mice/group). The data were pooled from five independent experiments. (D) CD1d expression on macrophages and dendritic cells (DCs) analyzed by flow cytometry (5 mice/group). The data were pooled from five independent experiments. (E) Intracellular IL‐12 levels in macrophages and DCs analyzed by flow cytometry (5 mice/group). The data were pooled from five independent experiments. Box plots: horizontal lines in boxes represent the medians, the boxes represent the interquartile ranges, and the whiskers extend to extreme values. Gray, isotype control; blue, water‐treated B6 mice; red, alcohol‐treated B6 mice. *p < .05, **p < .01; Mann–Whitney test. n.s., not significant

We found no statistical differences in bone cell populations between alcohol‐ and water‐treated B6 mice; therefore, we measured CD69 expression on CD4^+^ T cells, NK cells, and NKT‐like cells. CD69 is the activation marker of lymphocytes. Interestingly, CD69 expression on NK cells and NKT‐like cells was significantly decreased by alcohol consumption (Figure 3A). However, on CD4^+^ T cells, its expression was slightly decreased by alcohol treatment. These findings suggest that alcohol consumption could suppress lymphocyte activity.

Next, we evaluated intracellular IL‐4 production by CD4^+^ T cells, NK cells, and NKT‐like cells. As shown in Figure 3B, IL‐4 production was suppressed by alcohol consumption in NKT‐like cells. In contrast, IFN‐γ production by CD4^+^ T cells, NK cells, and NKT‐like cells were not different between the groups; however, IFN‐γ production by NKT‐like cells was slightly decreased by alcohol consumption (*p* = .067; Figure S6A). These results indicate that alcohol consumption can suppress the activity of NKT‐like cells, which are the most powerful producers of IL‐4. It is possible that alcohol can enhance osteoclastogenesis by inactivating NKT‐like cells. NKT cells can be divided functionally into Th1 and Th2 subsets based on the expression of the transcription factors T‐bet and GATA3, respectively. Therefore, we investigated the intracellular expression of T‐bet and GATA3 in NKT‐like cells in alcohol‐ and water‐treated B6 mice. As presented in Figure 3C, GATA3 expression was significantly decreased by alcohol consumption. GATA3 regulates CD4^+^ T‐cell differentiation into the Th2 lineage and regulates IL‐4 production.^34^, ^35^ Meanwhile, alcohol consumption was related to a slight decrease in the expression of T‐bet (Figure S6B), which modulates CD4^+^ T‐cell differentiation into the Th1 lineage and regulates IFN‐γ production.^36^ These findings support that alcohol consumption can suppress the differentiation of NKT‐like cells into Th2 subsets and inhibit IL‐4 secretion.

Next, we investigated the function of APCs in the bone because APCs are essential for the regulation of T, NK, and NKT cells. APCs can interact directly and indirectly with NKT cells via antigen presentation and cytokine secretion. We examined the expressions of CD86 and CD1d on macrophages and DCs in bone from alcohol‐ and water‐treated B6 mice. CD86 is considered a co‐stimulatory marker, and CD1d is considered a key molecule that presents glycolipid/lipid antigens to NKT cells.^21^ CD86 expression on macrophages and DCs was not altered by alcohol consumption (Figure S7); however, CD1d expression on macrophages and DCs was significantly decreased in alcohol‐treated B6 mice (Figure 3D). To address the functional alteration of APCs by alcohol consumption, intracellular IL‐12 production by macrophages and DCs was assessed. As shown in Figure 3E, IL‐12 production by macrophages and DCs was obviously diminished by alcohol consumption. As described previously, NKT cells can be activated by APCs via the CD1d–TCR axis and IL‐12 secretion.^37^ Therefore, we suppose that the alterations of CD1d expression and IL‐12 production on macrophages and DCs following alcohol consumption might change the function of NKT‐like cells in bone. Collectively, alcohol may reduce CD1d expression and IL‐12 production by APCs.

### 3.4 Alcohol‐induced osteoporosis developed independently of iNKT cells

To investigate whether NKT‐like or iNKT cells are essential for osteoporosis development, we evaluated the effects of alcohol in *Cd1d* ^−/−^ mice. In *Cd1d* ^−/−^ mice, NK1.1^+^CD3^+^ cells, but not iNKT cells, were present in the spleen, BM, and bone (Figure S8A). This indicates that NKT‐like cells are clearly present in *Cd1d* ^−/−^ mice similarly as observed in B6 mice. In *Cd1d* ^−/−^ mice, trabecular BMD was decreased by alcohol consumption similarly as observed in B6 mice (Figure S8B). This result indicates that NKT‐like cells play important roles in the onset of alcohol‐induced osteoporosis, whereas iNKT cells have only a marginal impact on its onset.

### 3.5 Administration of OCH improves alcohol‐induced osteoporosis with the enhancement of IL‐4 production of NKT‐like cells and iNKT cells

**Figure 4:**
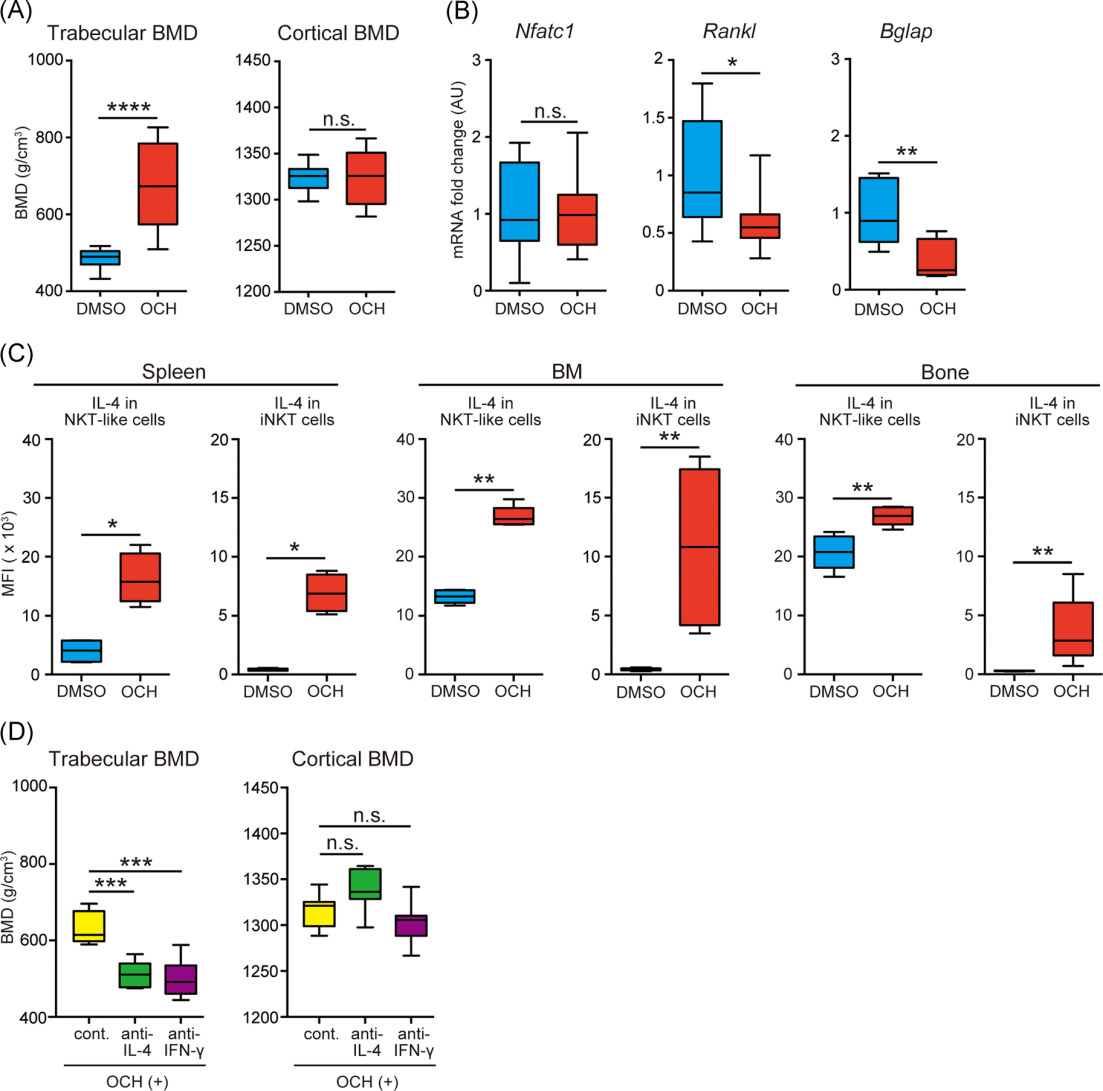
Administration of OCH suppresses alcohol‐induced osteoporosis in an IL‐4‐dependent manner. Alcohol‐treated B6 mice received intraperitoneal injections of OCH or vehicle every 48 h from 9 to 13 weeks of age. Mice were killed 24 h after the last intraperitoneal injection of OCH or vehicle. (A) Trabecular and cortical bone mineral density (BMD) measured in alcohol‐treated B6 mice that received OCH (red) or vehicle (blue) (8–10 mice/group). The data were pooled from eight independent experiments. (B) Messenger RNA expression of *Nfatc1*, *Rankl*, and *Bglap* in bone from alcohol‐treated B6 mice that received OCH (red) or vehicle (blue) (8–10 mice/group). The data were pooled from eight independent experiments. (C) Intracellular IL‐4 production by natural killer T (NKT)‐like cells and invariant natural killer T (iNKT) cells obtained from the spleen, bone marrow (BM), and bone of alcohol‐treated B6 mice that received OCH (red) or vehicle (blue) (5 mice/group). The data were pooled from four independent experiments. (D) Trabecular and cortical BMD in alcohol‐treated B6 mice that received OCH along with anti‐IL‐4 (green) and anti‐IFN‐γ (purple) neutralizing antibodies. Yellow, isotype matched IgG administration as the control. These antibodies were administered to alcohol‐treated B6 mice at the same time as OCH every 48 h (8–10 mice/group). The data were pooled from four independent experiments. Box plots: horizontal lines of boxes represent the medians, the boxes represent the interquartile ranges, and the whiskers extend to extreme values. *p < .05, **p < .01, ***p < .005, ****p < .001; Mann–Whitney *U* test. n.s., not significant

OCH is a glycolipid antigen and a ligand of iNKT cells that promotes IL‐4 secretion, and it has been tested in clinical trials for the treatment of Crohn's disease and multiple sclerosis.^38^ In the present study, IL‐4 production by NKT‐like cells was significantly decreased in mice with alcohol‐induced osteoporosis (Figure 3B). Hence, we checked whether OCH can prevent alcohol‐induced osteoporosis. Alcohol‐treated B6 mice received sequential intraperitoneal injections of OCH or vehicle every 48 h during alcohol consumption. Surprisingly, OCH administration improved trabecular BMD in alcohol‐treated B6 mice (Figure 4A). Meanwhile, *Rankl* and *Bglap* expression, but not *Nfatc1* expression, was decreased by OCH treatment (Figure 4B). To explain these beneficial effects of OCH, intracellular IL‐4 and IFN‐γ production by NKT‐like and iNKT cells in the spleen, BM, and bone was examined. As expected, IL‐4 production by iNKT cells was increased by OCH treatment (Figure 4C), as was IFN‐γ production (Figure S9). In addition, the production of these cytokines by NKT‐like cells was also enhanced by OCH treatment in the spleen, BM, and bone (Figures 4C and S9). Finally, to evaluate whether inhibition of IL‐4 and IFN‐γ counteracts the therapeutic effects of OCH, an anti‐IL‐4 or anti‐IFN‐γ neutralizing antibody was administered to alcohol‐treated B6 mice simultaneously with OCH every 48 h. IL‐4 and IFN‐γ blockade did not alter cortical BDM; however, these treatments counteracted the protective effects of OCH on trabecular BMD (Figure 4D). These results indicate that OCH can facilitate IL‐4 and IFN‐γ production by both iNKT and NKT‐like cells in the systemic and local milieu. Collectively, OCH can prevent the decrease of BMD induced by alcohol consumption via the stimulation of iNKT and NKT‐like cells. We propose that alcohol‐induced osteoporosis develops independently of iNKT cells, but the activation of iNKT and NKT‐like cells plays a role in the alleviation of alcohol‐induced osteoporosis.

## 4 DISCUSSION

In this study, we first observed that NKT‐like cells largely exist in the bone as opposed to the BM and spleen. In addition, NKT‐like cells produce more IL‐4 than CD4^+^ T and NK cells. Meanwhile, alcohol consumption suppresses the immunostimulatory activity of macrophages and DCs, leading to the suppression of IL‐4 production by NKT‐like cells with a significant decrease of BMD and upregulation of *Nfatc1* and *Rankl*. Furthermore, OCH can prevent alcohol‐induced osteoporosis by stimulating IL‐4 and IFN‐γ production by both NKT‐like and iNKT cells. These results might provide new perspectives in osteoimmunology, in particular, alcohol‐induced osteoporosis.

Previously, the stimulation of osteoclastogenesis was reported to be accompanied by decreases in trabecular BMD in alcohol‐treated mice.^39^ In general, the differentiation and proliferation of osteoclasts can be suppressed by IL‐4.^15^, ^16^ Our present results that IL‐4 production by NKT‐like cells was suppressed in mice with alcohol‐induced osteoporosis support these findings. IL‐4 is a Th2 cytokine produced by helper T cells; however, we found that NKT‐like cells more strongly produced IL‐4 than CD4^+^ T cells and NK cells. These results suggest that NKT‐like cells play a key role in regulating the activity of osteoclasts. IFN‐γ is also considered to have suppressive effects on osteoclast and osteoclast progenitor activity.^18^ Indeed, we found that alcohol treatment slightly decreased IFN‐γ production by NKT‐like cells, whereas IFN‐γ production by immune cells was remarkably elevated by the administration of OCH. The downregulation of IFN‐γ, as well as IL‐4, may contribute to the promotion of osteoclastogenesis by CAC.

In this study, we proposed the mechanism of alcohol‐induced osteoporosis from an immunological perspective. In particular, alcohol treatment suppresses the immunostimulatory activity of APCs, leading to the downregulation of CD1d expression and IL‐12 production. Consequently, IL‐4 production by NKT‐like cells, which negatively regulate osteoclast differentiation and proliferation, may be diminished, thereby inducing excessive activation of osteoclasts and subsequently osteoporosis. The precise mechanism of the downregulation of the immunostimulatory activity of APCs in alcohol‐treated mice is unclear. We suggest that the inhibition of APCs may be attributable to the alteration of lipid metabolism in BM by alcohol. In general, BMMSCs can differentiate into osteoblasts and adipocytes.^40^, ^41^ A study suggested that peroxisome proliferator‐activated receptor γ, a key regulator of both adipogenesis and osteogenesis that is controlled by NO through the denitrosylation activity of GSNOR/ADH3, balances the differentiation of BMMSCs into adipocytes or osteoblasts.^42^, ^43^ Furthermore, CAC can induce BMMSC differentiation toward adipocytes^8^ and reduce osteoclast generation via activation of the mTOR pathway.^13^ These findings suggest that alcohol consumption modulates fat accumulation in BM.

APCs and NKT cells can be activated by glycolipid/lipid antigens originating from the fat tissues.^29^ Glycolipid/lipid antigens are captured by APCs, and they establish the immunostimulatory activity for other immune cells including NKT cells. A recent report revealed that the glycolipid antigen β‐GluCer can bind the Mincle receptor and facilitate the immunostimulatory activity of APCs.^44^ In addition, many types of endogenous glycolipid antigens can be captured by APCs, leading to the stimulation of iNKT cells.^45^ These findings suggest that the alteration of glycolipid/lipid antigens in BM may have some relevance to the function of APCs. We suppose that CAC can increase adipose tissue content in BM; however, the glycolipid/lipid components that effectively activate the APCs–NKT‐like cell axis may be suppressed by alcohol consumption. Further studies are in progress to determine the glycolipid/lipid antigens that are altered by alcohol treatment in bone, and understanding the alterations of glycolipid/lipid antigens in BM by alcohol may clarify the mechanism underlying osteoporosis related to the APCs–NKT‐like cell axis.

RANKL production is primarily dependent on osteocytes rather than osteoblasts,^46^ and CAC induced massive osteocyte apoptosis accompanied by lipid accumulation within the bone matrix.^47^ Our result demonstrated that OCH treatment suppressed the *Rankl* and *Bglap* expression with a consequent increase in trabecular BMD, suggesting that *Rankl* upregulation was caused by the direct effects of alcohol on the bone matrix, leading to the disturbance of the osteoblast–osteoclast balance representing low turnover bone metabolism and increasing trabecular BMD, similar to the effects of the anti‐RANKL antibody denosumab.^48^ Conversely, *Nfatc1* expression was not statistically changed by OCH treatment. Studies have reported RANKL‐independent pathways that promote osteoclastogenesis^49^ and that IL‐6 signaling can induce the upregulation of c‐Fos, expression leading to *Nfatc1* expression.^50^ Regarding NKT cell differentiation, NFATc1 is also essential for their development and maturation by activating early growth response 2 via the calcineurin–NFAT signaling pathway.^51^ Furthermore, *Nfatc1* inactivation could interfere with IL‐4 secretion.^52^ These reports indicate that *Nfatc1* expression via RANKL‐independent pathways might be stimulated by OCH treatment, resulting in the development of NKT‐like cells and iNKT cells with significant IL‐4 production. Thus, OCH might improve alcohol‐induced osteoporosis.

iNKT cells exhibit various immune reactions depending on the stimulating antigen.^21^, ^45^, ^53^ α‐GalCer, which was originally identified in marine sponges, can induce the rapid production of numerous cytokines, including IFN‐γ, IL‐4, and TNF‐α, by iNKT cells.^54^ A previous study suggested that α‐GalCer could induce osteoclastogenesis via TNF‐α, which overcomes the suppressive effect of IFN‐γ.^55^ Alternatively, OCH, which is an α‐GalCer analog, increases IL‐4 production rather than IFN‐γ production by iNKT cells.^38^ Indeed, we found that OCH treatment could stimulate the iNKT and NKT‐like cells, which are powerful sources of IL‐4 production, in the systemic and local milieu, and also prevent the alcohol‐induced decrease of BMD in B6 mice. Recent research suggested that the antigenic lipid α‐hexosylceramide from cow's milk could be recognized by the CD1d–T‐cell receptor interface.^56^ This result implies that the oral intake of cow's milk has beneficial effects on both calcium intake and activation of the APC–NKT‐like cell axis. Various drugs, foods, and herbal medicines contain several undefined glycolipid antigens; these agents may be useful for the prevention and treatment of alcohol‐induced bone loss and osteoporosis.

To date, many treatments, including vitamin D,^57^ bisphosphonate,^58^ and alendronate,^59^ have been considered suitable candidates for the treatment of alcohol‐induced osteoporosis. We propose that glycolipid antigens such as OCH are also useful candidates for this indication. The regulation of innate immune cells, macrophages, DCs, NKT‐like cells, and iNKT cells could represent a new therapeutic approach for preventing alcohol‐induced osteoporosis.

## CONFLICT OF INTERESTS

The authors declare that there are no conflict of interests.

## ETHICS STATEMENT

All animal experiments were conducted in compliance with the protocol reviewed by the Institutional Committee of Laboratory Animals and approved by the President of the Nippon Medical School (approval number 28‐001, 27‐184), and the experiments were conducted according to the Guidelines for the Care and Use of Laboratory Animals issued by the National Institutes of Health (Bethesda, MD, USA).

## AUTHOR CONTRIBUTIONS

Munehiro Naruo and Yasuyuki Negishi designed the study. Munehiro Naruo, Midori Katsuyama, and Takahisa Okuda established the murine alcohol consumption model; Munehiro Naruo performed the RT‐qPCR, qCT, and μCT studies; Munehiro Naruo and Yasuyuki Negishi performed the TRAP staining; Yasuyuki Negishi performed the flow cytometry study; Munehiro Naruo, Yasuyuki Negishi, and Rimpei Morita participated in data interpretation. Munehiro Naruo and Yasuyuki Negishi wrote the first draft of the manuscript; Yasuyuki Negishi and Rimpei Morita wrote the final version of the manuscript; Ken Okazaki and Rimpei Morita provided scientific insight and supervised the study. All authors revised the manuscript, approved the manuscript to be published, and agree to be accountable for all aspects of the work in ensuring that questions related to the accuracy or integrity of any part of the work are appropriately investigated and resolved.

## Supporting information

Supplementary information.

## Data Availability

The data that support the findings of this study are available from the corresponding authors upon reasonable request.
