# Supplementary material for: Alcohol consumption induces murine osteoporosis by downregulation of natural killer T‐like cell activity
Source: Immun Inflamm Dis. 2021 Jul 2;9(4):1370–82. doi: 10.1002/iid3.485 (PMC8589379; doi:10.1002/iid3.485)
Supplement: Supplementary file 1 — Supplementary information. [file IID3-9-1370-s001.pdf]

Supporting Information for

Alcohol consumption induces murine osteoporosis by downregulating natural killer T-like cell activity

Munehiro Naruo, Yasuyuki Negishi, Takahisa Okuda, Midori Katsuyama, Ken Okazaki, and Rimpei Morita

Corresponding authors

Yasuyuki Negishi, M.D., Ph.D.
Email: negi@nms.ac.jp

Rimpei Morita, M.D., Ph.D.
Email: rimpei-morita@nms.ac.jp

This PDF file includes:

Supporting Information Figures S1 to S9
Supporting Information Tables S1 to S2

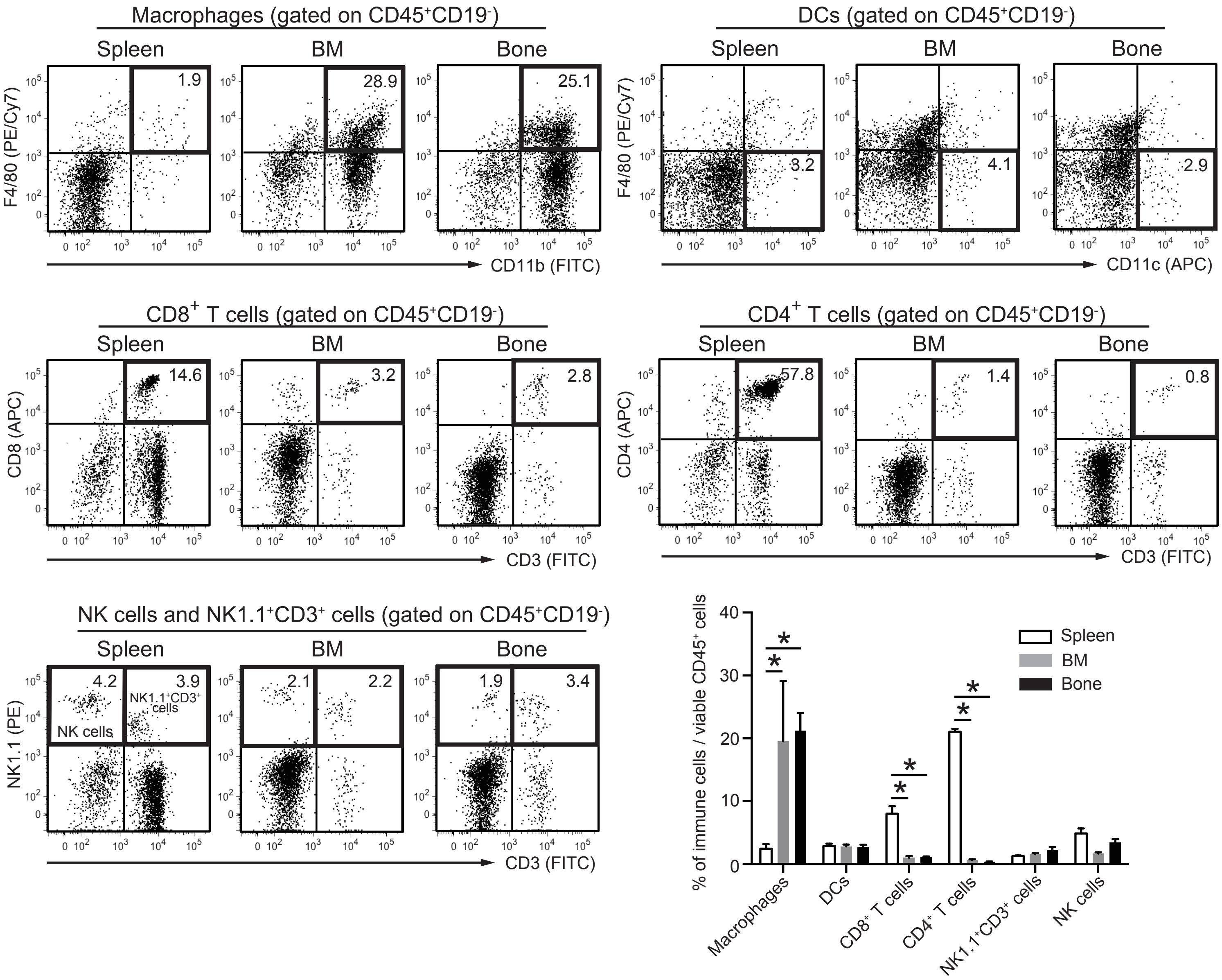

Supporting Information Figure S1. Identification and distribution of immune cells in the spleen, BM, and bone. Representative flow cytometric analysis of macrophages, DCs, CD8$^+$ T cells, CD4$^+$ T cells, NK cells, and NK1.1$^+$CD3$^+$ cells and the percentages of these cells among viable CD45$^+$ cells in the spleen, BM, and bone. The full gating strategies are presented in Supporting Information 2 (bone), 3 (BM), and 4 (spleen). Data represent the mean ± S.D. (7 mice/group), and were pooled from six independent experiments. *$P < 0.05$; one-way ANOVA followed by Bonferroni post-tests.

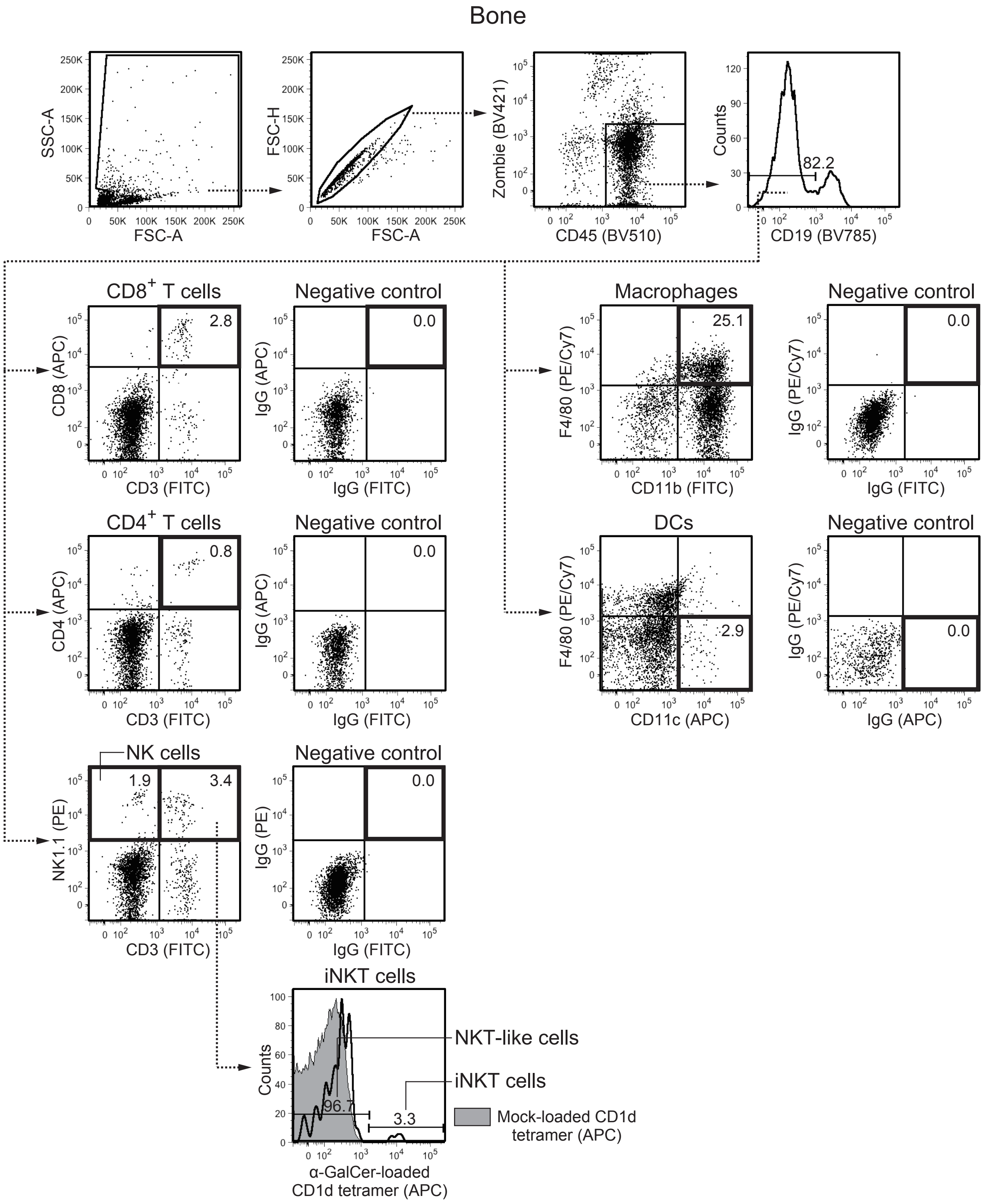

Supporting Information Figure S2. Representative gating strategies of flow cytometric analysis for the detection of macrophages, DCs, CD8⁺ T cells, CD4⁺ T cells, NK cells, iNKT cells, and NKT-like cells in bone. Macrophages were identified as Zombie⁻, CD45⁺, CD19⁻, F4/80⁺, and CD11b⁺. DCs were identified as Zombie⁻, CD45⁺, CD19⁻, F4/80⁻, and CD11c⁺. CD8⁺ T cells were identified as Zombie⁻, CD45⁺, CD19⁻, CD3⁺, and CD8⁺. CD4⁺ T cells were identified as Zombie⁻, CD45⁺, CD19⁻, CD3⁺, and CD4⁺. NK cells were identified as Zombie⁻, CD45⁺, CD19⁻, CD3⁻, and NK1.1⁺. iNKT cells were identified as Zombie⁻, CD45⁺, CD19⁻, CD3⁺, NK1.1⁺, and α-GalCer–loaded CD1d tetramer⁺. NKT-like cells were identified as Zombie⁻, CD45⁺, CD19⁻, CD3⁺, NK1.1⁺, and α-GalCer–loaded CD1d tetramer⁻.

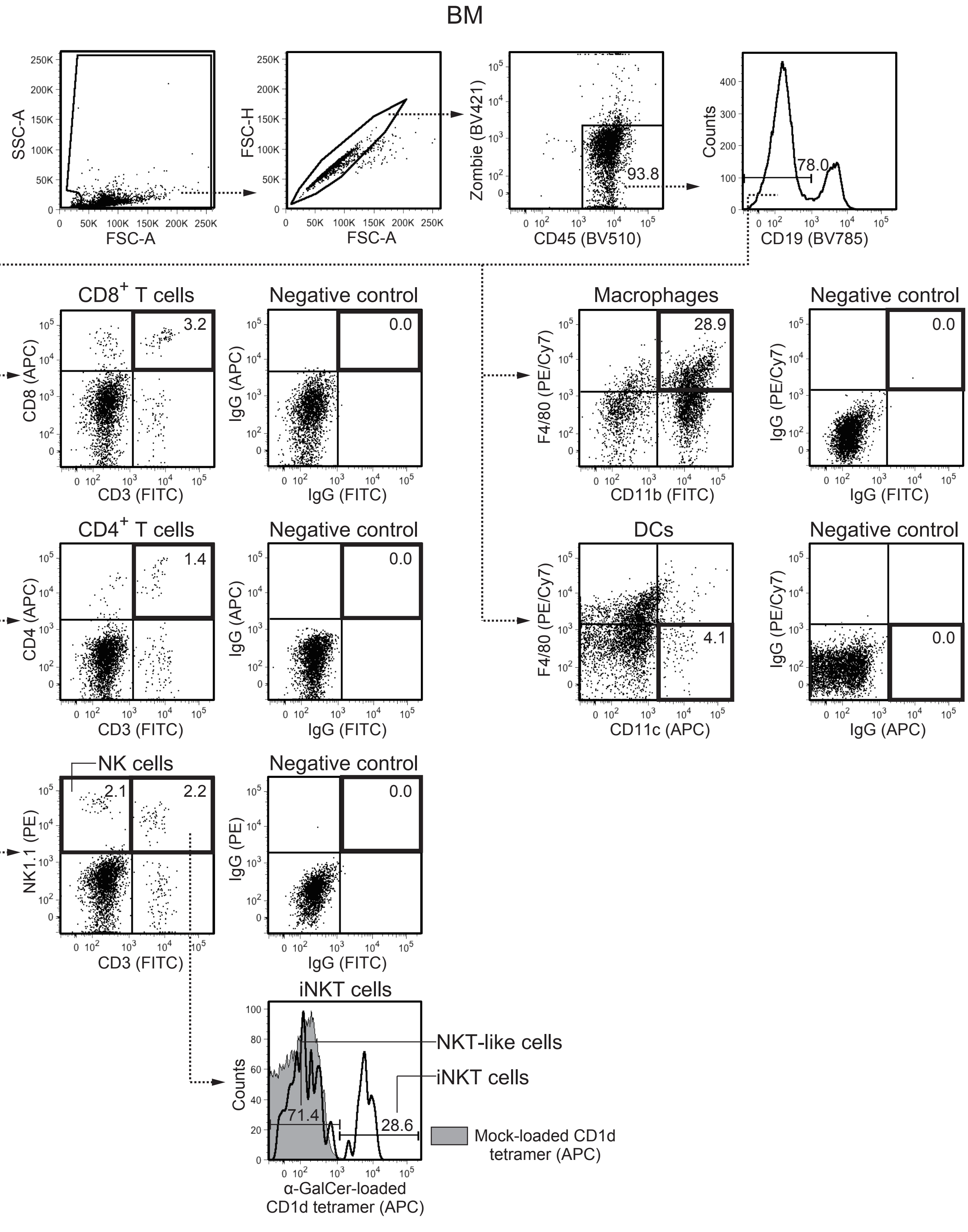

Supporting Information Figure S3. Representative gating strategies of flow cytometric analysis for the detection of macrophages, DCs, CD8⁺ T cells, CD4⁺ T cells, NK cells, iNKT cells, and NKT-like cells in BM. Macrophages were identified as Zombie⁻, CD45⁺, CD19⁻, F4/80⁺, and CD11b⁺. DCs were identified as Zombie⁻, CD45⁺, CD19⁻, F4/80⁻, and CD11c⁺. CD8⁺ T cells were identified as Zombie⁻, CD45⁺, CD19⁻, CD3⁺, and CD8⁺. CD4⁺ T cells were identified as Zombie⁻, CD45⁺, CD19⁻, CD3⁺, and CD4⁺. NK cells were identified as Zombie⁻, CD45⁺, CD19⁻, CD3⁻, and NK1.1⁺. iNKT cells were identified as Zombie⁻, CD45⁺, CD19⁻, CD3⁺, NK1.1⁺, and α-GalCer–loaded CD1d tetramer⁺. NKT-like cells were identified as Zombie⁻, CD45⁺, CD19⁻, CD3⁺, NK1.1⁺, and α-GalCer–loaded CD1d tetramer⁻.

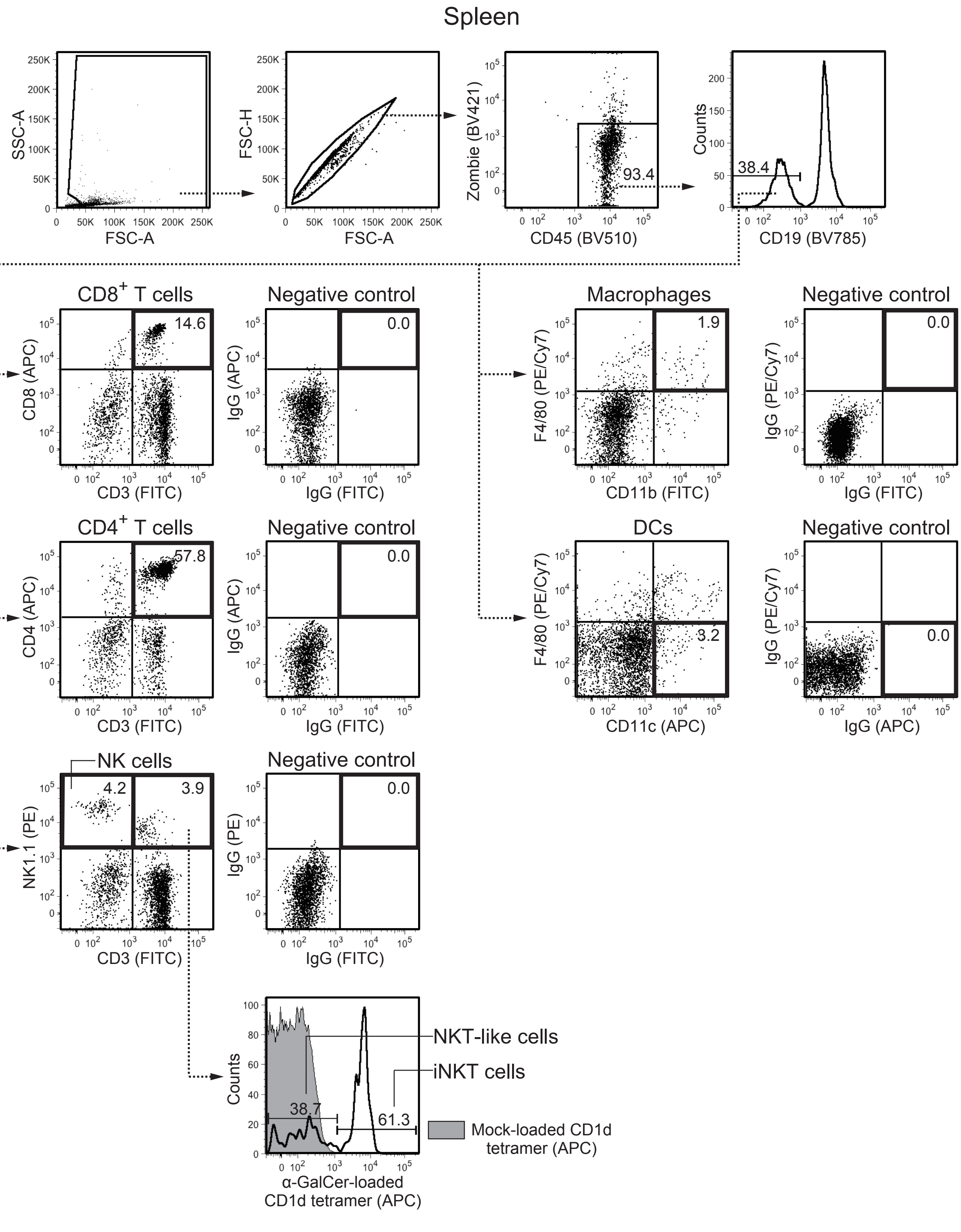

Supporting Information Figure S4. Representative gating strategies of flow cytometric analysis for the detection of macrophages, DCs, CD8⁺ T cells, CD4⁺ T cells, NK cells, iNKT cells, and NKT-like cells in spleen. Macrophages were identified as Zombie⁻, CD45⁺, CD19⁻, F4/80⁺, and CD11b⁺. DCs were identified as Zombie⁻, CD45⁺, CD19⁻, F4/80⁻, and CD11c⁺. CD8⁺ T cells were identified as Zombie⁻, CD45⁺, CD19⁻, CD3⁺, and CD8⁺. CD4⁺ T cells were identified as Zombie⁻, CD45⁺, CD19⁻, CD3⁺, and CD4⁺. NK cells were identified as Zombie⁻, CD45⁺, CD19⁻, CD3⁻, and NK1.1⁺. iNKT cells were identified as Zombie⁻, CD45⁺, CD19⁻, CD3⁺, NK1.1⁺, and α-GalCer–loaded CD1d tetramer⁺. NKT-like cells were identified as Zombie⁻, CD45⁺, CD19⁻, CD3⁺, NK1.1⁺, and α-GalCer–loaded CD1d tetramer⁻.

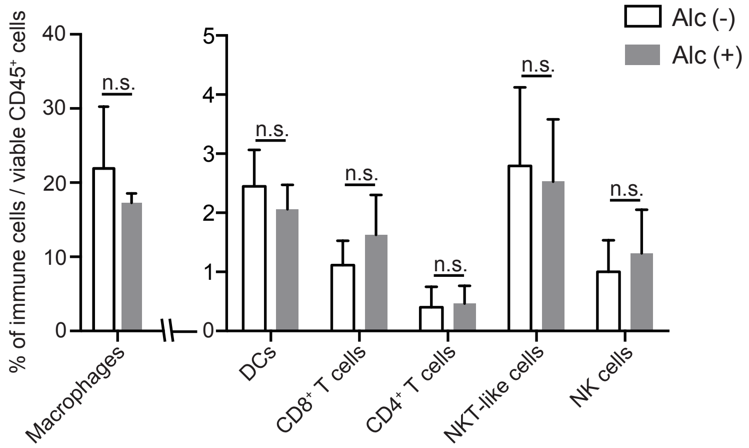

Supporting Information Figure S5. Alcohol consumption does not alter the distribution of immune cells in bone. The percentage of macrophages, DCs, CD8$^+$ T cells, CD4$^+$ T cells, NK cells, and NKT-like cells among viable CD45$^+$ cells in bone obtained from alcohol- and water-treated B6 mice. NK1.1$^+$CD3$^+$ cells were defined as NKT-like cells. The femurs and tibiae were separated, and cells in these bones were analyzed via flow cytometry. Data represent the mean ± SD (6–8 mice/group), and were pooled from six independent experiments. Mann–Whitney test. n.s.: not significant.

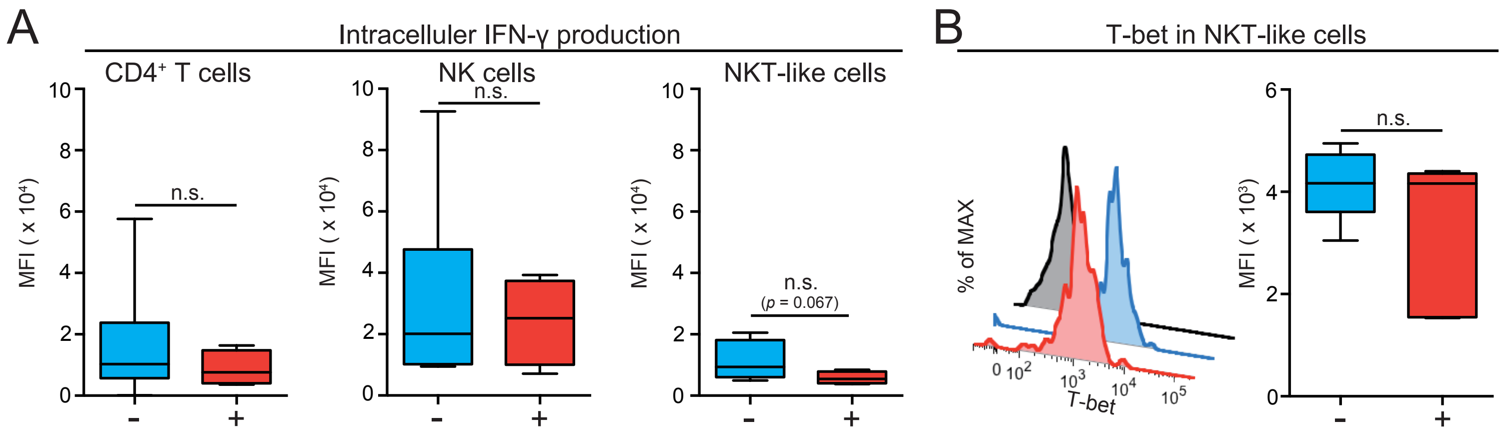

Supporting Information Figure S6. Alcohol consumption does not alter the intracellular IFN-γ production in immune cells. (A) Intracellular IFN-γ levels in $CD4^+$ T cells, NK cells, and NKT-like cells in bone analyzed via flow cytometry (5–6 mice/group). The data were pooled from five independent experiments. (B) Expression of T-bet in NKT-like cells analyzed by flow cytometry (5 mice/group). The data were pooled from five independent experiments. Grey: isotype control. Blue: water-treated B6 mice. Red: alcohol-treated B6 mice. Box plots: horizontal lines of boxes represent the medians, the boxes represent the interquartile ranges, and the whiskers extend to extreme values. Mann-Whitney test. n.s.: not significant.

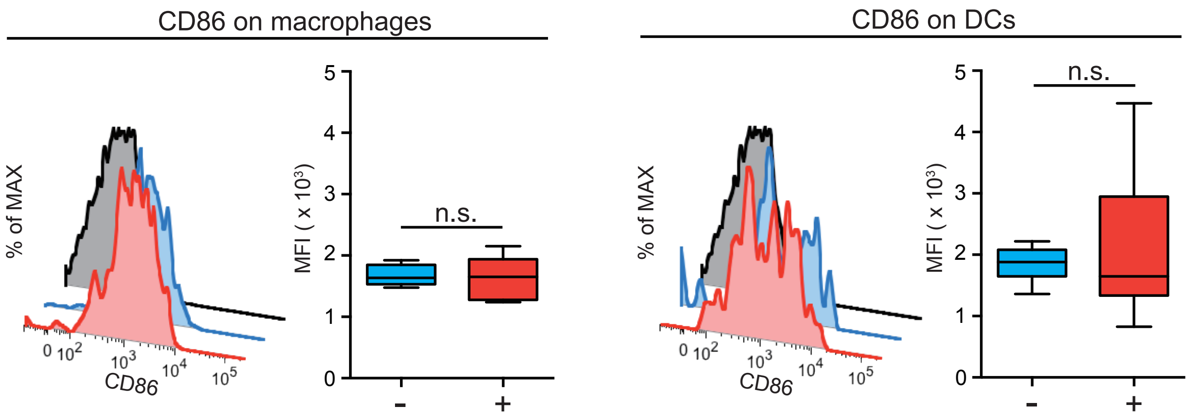

Supporting Information Figure S7. Alcohol consumption does not alter the CD86 expression levels on APCs. CD86 expression on macrophages and DCs was analyzed by flow cytometry. Grey: isotype control. Blue: water-treated B6 mice. Red: alcohol-treated B6 mice. Box plots: horizontal lines of boxes represent the medians, the boxes represent the interquartile ranges, and the whiskers extend to extreme values (5 mice/group). The data were pooled from five independent experiments. Mann–Whitney test. n.s.: not significant.

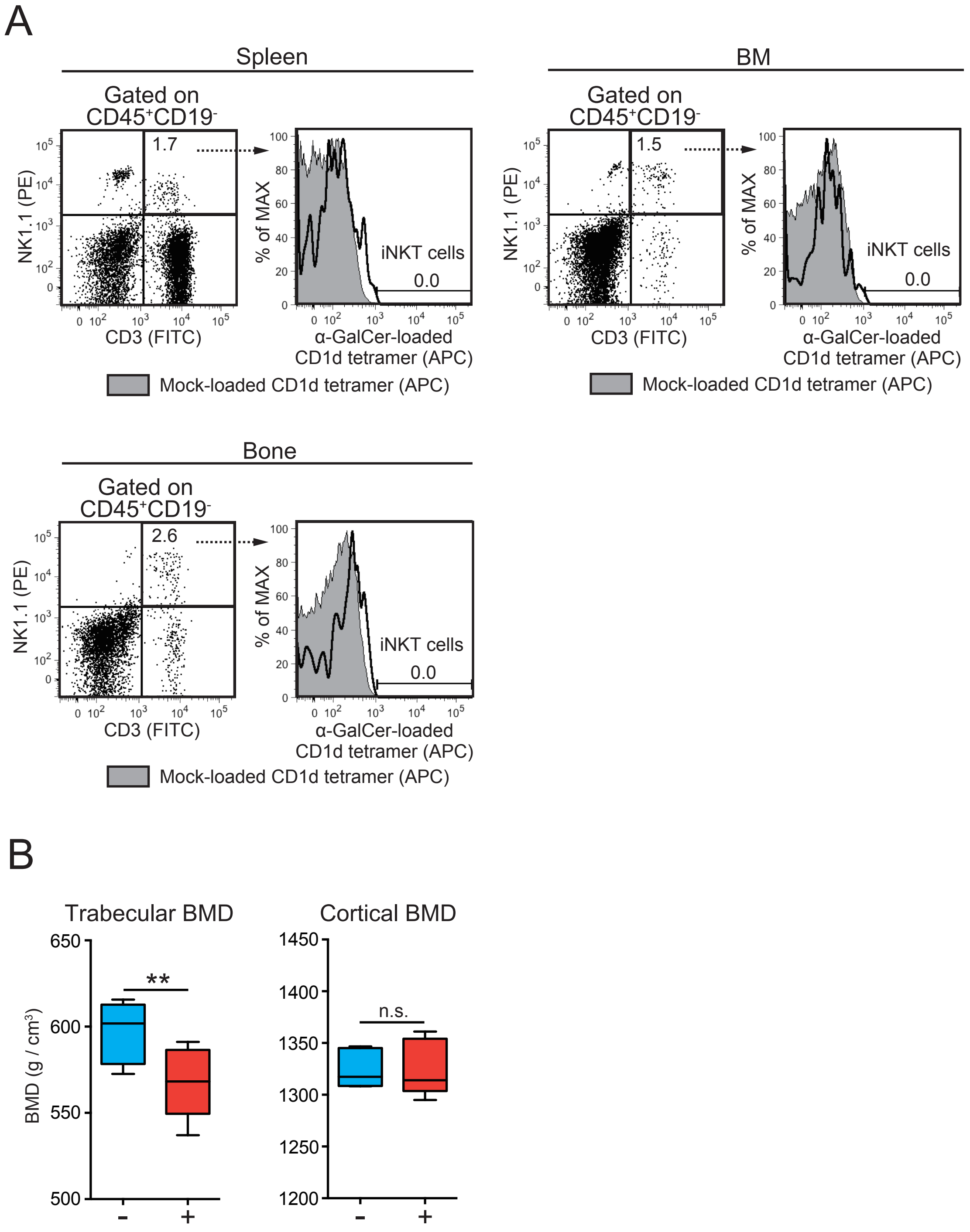

Supporting Information Figure S8. Alcohol-induced osteoporosis is promoted in iNKT cell-deficient (Cd1d$^{-/-}$) mice, which possess NKT-like cells. (A) Representative flow cytometry of NK1.1^{+}CD3^{+} cells and CD1d tetramer-positive cells among NK1.1^{+}CD3^{+} cells in the spleen, BM, and bone obtained from Cd1d$^{-/-}$ mice. (B) Trabecular and cortical BMD in alcohol-treated (red) and water-treated (blue) Cd1d$^{-/-}$ mice. Box plots: horizontal lines of boxes represent the medians, the boxes represent the interquartile ranges, and the whiskers extend to extreme values (6 mice/group). The data were pooled from four independent experiments. *$P < 0.05$; Mann–Whitney test. n.s.: not significant.

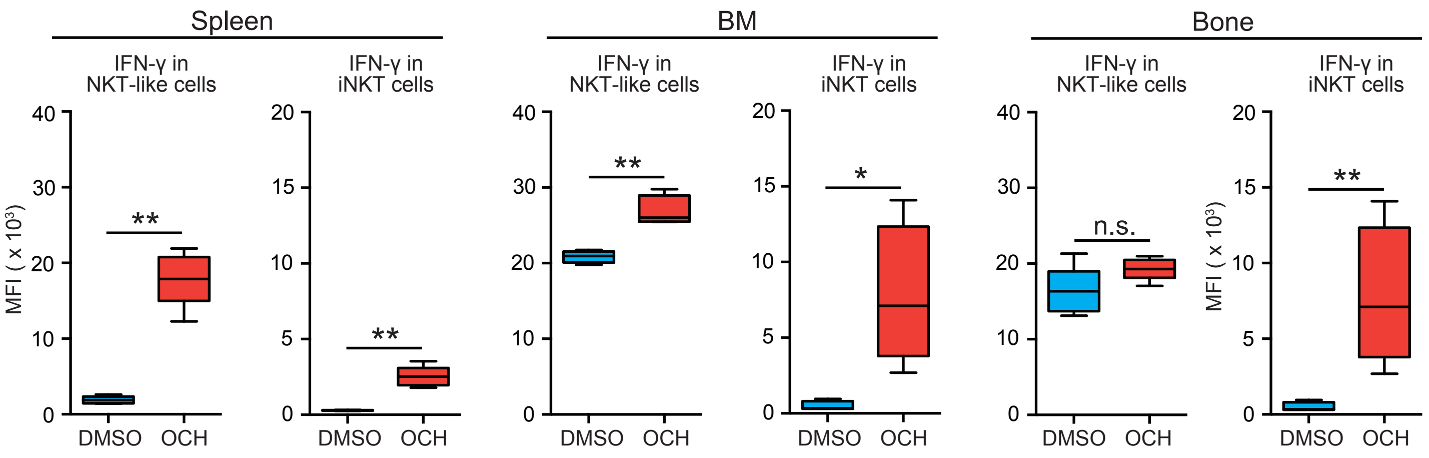

Supporting Information Figure S9. Administration of OCH enhances IFN-γ production by NKT-like cells and iNKT cells obtained from the spleen, BM, and bone of alcohol-treated B6 mice. Alcohol-treated B6 mice received i.p. injections of OCH (red) or vehicle (blue) every 48 h from 9 to 13 weeks of age. Mice were killed 24 h after the last i.p. injection of OCH or vehicle. Intracellular IFN-γ production by NKT-like cells and iNKT cells were analyzed by flow cytometry (5 mice/group). The data were pooled from four independent experiments. Box plots: horizontal lines of boxes represent the medians, the boxes represent the interquartile ranges, and the whiskers extend to extreme values. *P < 0.05, **P <0.01; Mann–Whitney test. n.s.: not significant.

Supporting Information Table S1. Primer used for RT-qPCR

Gene	Forward primer	Reverse primer	Accesion no.
Nfatc1	CCCGTCACATTCTGGTCCAT	CAAGTAACCGTGTAGCTGCACAA	NM_01679.41
Rankl	TGAAGACACACTACCTGACTCCTG	CCCACAATGTGTTGCAGTTC	NM_011613.3
Bglap	AAGCAGGAGGGCAATAAGGT	GTTCTGGAGAGCAGCCAAAG	NM_001305448.1
Actb	GCGCAAGTTAGGTTTTGTCAAAG	TGGATCAGCAAGCAGGAGTAC	NM_007393.5

Nfatc1; nuclear factor of activated T cells, cytoplasmic, calcineurin dependent 1, *Rankl*; receptor activator of nuclear factor kappa B ligand, *Bglap*; bone gamma-carboxyglutamate protein, *Actb*; Actin beta

Supporting Information Table S2. Antibodies used in the study

Antibody	Clone	Source
CD3ε-FITC	145-2C11	BioLegend (San Diego, CA)
CD4-APC	RM4-5	BioLegend
CD8-APC	53-6.7	BioLegend
CD11b-FITC	M1/70	BioLegend
CD11c-APC	N418	BioLegend
CD19-BV785	6D5	BioLegend
CD45-BV510	30-F11	BioLegend
CD69-BUV395	H1.2F3	BD OptiBuild (San Jose, CA)
CD86-PE	GL-1	BioLegend
NK1.1-PE	PK136	BioLegend
F4/80-PE/Cy7	BM8	BioLegend
CD1d-BUV395	1B1	BD OptiBuild
α-GalCer loaded CD1d-tetramer-APC	code:E001-4B	Proimmune (Oxford, UK)
Mock loaded CD1d-tetramer-APC	code:E002-4A	Proimmune
IL-4-PE/Cy7	11B11	BD Pharmingen (San Jose, CA)
IFN-γ-PE/Cy7	XMG1.2	BD Pharmingen
IL-12/IL-23p40-PE/Cy7	C17.B	Invitrogen (Carlsbad, CA)
T-bet-PE	REA102	Miltenyi Biotec (San Diego, CA)
GATA3-PE	REA174	Miltenyi Biotec
REA control (I)-PE	REA293	Miltenyi Biotec
